# Supplementary material for: Acceptance and compliance with micronutrient powder (MNP) among children aged 6–23 months in northern Nigeria
Source: PLOS Glob Public Health. 2022 Oct 17;2(10):e0000961. doi: 10.1371/journal.pgph.0000961 (PMC10022258; doi:10.1371/journal.pgph.0000961)
Supplement: S1 Dataset — (ZIP) [file pgph.0000961.s008.zip › S8_Dataset/Spot Checks.zip!/Spot Checks/03072015_A_SO_AD_1_SC.pdf]

# SPOT CHECK RECORDING FORM

**State: Adamawa _ LGA:Song___________ Community: modungo_**

**Name of caregiver (female): …Binta Abubakar…………….**

**Name of caregiver (male): ……………………………………_**

**Sex of index child: Male_____ Age of index child (in months): 12month________**

## Spot Check Information

1. **Date of MNP distribution to household** (circle option below based on LGA name above)**:**
   - **Kebbi:** BirninKebbi&Jega LGA: **June 16**
   - **Kebbi:** Danko-wasagu: **June 17**
   - **Adamawa:**Song, Ganye: **June 18**
   - **Adamawa:**Guyuk **June 19**
2. **Date of Spot Check Observation: _03/07/2015________________**
3. **Number of sachets remaining**(Ask the caregiver to show you and count the specific number of sachets remaining)**:**

   43

4. **Number of sachets that <u>should remain</u>using one MNP sachet per day** (Analyst only)**:**

   46

5. **Number of sachets remaining / number of sachets should be remaining**(Analyst only)**:**

   **____43_____ / _46________ = ____93.5_____ %**

## Probes

**Question 1.** You have **XX** MNP sachets remaining. Explain how easy or difficult it was to use the MNP product on a daily basis for your child 6 – 23 months? (Circle one answer)

**Very Difficult** **Difficult** **Easy** **Very Easy**

**Question 2.**Describe any specific challenges that you faced that made the MNP difficult to use on a daily basis?

There was no specific challenge that the child had refused to take it at first but forcefully the MNP

**Question 3.** Describe specific aspects of the MNP product that you or your child enjoyed that made it easier to use on a daily basis?

I have seen some improvement in my child.
His appetite has increased he eat more food now
He consumes more breast milk since he start taking MNP
He is healthier and stronger

**Question 4.** What questions do you have about the MNP based on your experience using it?

Is vomiting also a side effect of the MNP
Can we administer it for more than 60day if it is missed for a day

**Question 5.** What specific recommendations do you have that we can do as a program to make the MNP easier to use on a daily basis?

The recommendation is to keep up with this good work
Also continue to enlighten caregivers on the MNP sometimes like reminder.
